# How Should We Prepare Ourselves for New Normal Related to Stopped Competitions? Public Health and Athletes

**DOI:** 10.18502/ijph.v49i10.4714

**Published:** 2020-10

**Authors:** Amirhossein MEMARI, Ardalan SHARIAT, Joshua A. CLELAND

**Affiliations:** 1. Sports Medicine Research Center, Neuroscience Institute, Tehran University of Medical Sciences, Tehran, Iran; 2. Department of Public Health and Community Medicine, Tufts University School of Medicine, Boston, Mass, USA

## Dear Editor-in-Chief

Covid-19 has disrupted normal life not only among the general population and their daily activities, but also in different professions. Recreational and elite athletes have been ordered to halt their exercise activities. Currently there is no social physical activity or professional competitions, thus there will be huge financial consequences related to stopping those activities. Athletes will lose their physical and emotional fitness and sponsors could potentially lose their business (1–3).

What will be the new normal? What happens when athletes can do their training exercises at in home or indoor gyms or stadiums and sponsors can sell their tickets? Nevertheless, we do not know how long we must wait for this new normal life and what we can do now?

Before we reach the new normal, we recognize the very serious issues in the current situation. Staying at home, lack of social activities and physical activity, not only affect physical condition, but also the psychological condition of individuals. If a correct solution is not identified many individuals may not recover (4–6).

As a result of the absence of physical activity and lack of professional exercise training among elite athletes, we may need to learn how to replace international competitions with lower level of competitions in small societies or at the national level (4,6). This strategy will potentially keep individuals at a better level of physical fitness in comparison to waiting for an undetermined period of time. Certainly, recreational athletes or normal individuals who need physical activity for maintaining health can simply do stretching training combined with aerobic (dancing/walking) and resistance training (using body weight/water bottles) (7). If individuals do the suggested physical activities at home it will assist with overall health both physically and mentally (1,4).

You may not notice the psychological effects of your mind and performance. Most likely you will adapt to this situation, but what would be the level of your performance? Maybe playing web-based games or reading a book with motivational and challenging aims could be useful.

The possible solutions (for new normal) might include some of the following cases: new research to find the financial compensations; the medical approaches (PCR, rapid test,…) to screen spectators and keep them safe during a protected competition, age restrictions, new national competitions more than international ones … new regulations for leagues, athletes, sponsors and other stakeholders.
